# Pharmacokinetic and pharmacodynamic integration and resistance analysis of cefquinome against *Streptococcus uberis in vitro* dynamic model

**DOI:** 10.3389/fmicb.2025.1533892

**Published:** 2025-07-23

**Authors:** Chongyang Li, Junli Wang, Fanxi Guo, Fengyichi Zhang, Baochang Chen, Zihan Wang, Di Cao, Zugong Yu

**Affiliations:** Laboratory of Veterinary Pharmacology and Toxicology, Division of Basic Veterinary Medicine, College of Veterinary Medicine, Nanjing Agricultural University, Nanjing, China

**Keywords:** pharmacokinetic, pharmacodynamic, simulation, dynamic model, single high dosage, multiple low-dosage

## Abstract

**Introduction:**

*Streptococcus uberis (S. uberis)* is a major pathogen that causes acute clinical mastitis and its recurrent episodes in dairy cows.

**Methods:**

In this study, a peristaltic pump one-compartment open model was established to investigate the relationship between the pharmacokinetic and pharmacodynamic (PK/PD) indices of cefquinome (CFQ) against *S. uberis*. Bactericidal effects of single high-dosage versus multiple low-dosage administrations within the same drug dosage and best-fit dosage were assessed.

**Results:**

Static time-killing curves showed that the population of *S. uberis* was not changed when the drug concentration was below 1 × MIC. The maximum antibacterial effect was observed at 24 h, when the concentration exceeded 2 × MIC, showing a reduction by 5.73 log_10_ (CFU/mL), and the maximum kill rate was 0.22 h^−1^. *S. uberis* were cleared at 120 h when the concentration was ≥1 mg/L within single high-dosage groups, except for the 0.28 and 0.5 mg/L groups. The multiple-dose groups decreased below 2.22 log_10_ (CFU/mL) at 48 h and increased to 9 log_10_ (CFU/mL) at 120 h, but the group of 0.25 mg/L (4, q24) increased at 144 h. As the frequency of administration increased, the lag time increased following a population decline. The correlation coefficients between AUC_0-72h_/MBC, %T > MBC, and the antibacterial effects were 0.90 and 0.99%, respectively. %T > MBC was the best-fit PK/PD parameter of CFQ against *S. uberis*. The MIC of S1–S5 strains ranged from 0.0156–0.0625 μg/mL, and biofilm formation ability increased.

**Discussion:**

In conclusion, CFQ showed good efficacy and time-dependence. This study provides a reference for optimizing CFQ administration in *S. uberis*.

## 1 Introduction

*Streptococcus uberis* is a prevalent pathogen responsible for mastitis in dairy cows (Cobirka et al., 2020). *S. uberis* exhibits a spherical shape with a diameter of 0.5–2 μm and typically appears in pairs or chains (Chin et al., 1992). This bacterium typically appears alpha-hemolytic and is capable of causing the lysis of red blood cells, although it may also demonstrate non-hemolytic behavior under certain circumstances (Wente et al., 2019). *S. uberis* has been identified in various infected cows, including lactating, dry, heifer, and multiparous cows since the pathogen was initially isolated in 1932 (Busanello et al., 2017). *S. uberis* is the most common environmental pathogen that induces acute mastitis and recurrent infections (Sharun et al., 2021). *S. uberis* can adhere to and invade mammary epithelial cells and form biofilms and capsules, causing chronic infections that are difficult to effectively eradicate (Fessia and Odierno, 2021). Bovine mastitis has a significant effect on the health and economic productivity of cows. Berry’s estimation suggests that mastitis in a single dairy cow costs an average of £131 per year, accounting for 38% of the total cost of dairy cow production (Berry et al., 2004). Environmental changes, such as variations in temperature, humidity, ventilation, bedding, and feed conditions, can cause *S. uberis* to transition into pathogenic bacterium, triggering mastitis relapse (Gruet et al., 2001). Antibiotic treatment is the most commonly used therapeutic approach. Cefquinome (CFQ) is the first animal-specific, fourth-generation cephalosporin antibiotic available against *S. uberis* (Thomas et al., 2006).

The pharmacokinetic and pharmacodynamic (PK/PD) model has been extensively employed for optimizing antibiotic dosage and plays a crucial role in veterinary medicine, significantly contributing to the reduction of antibiotic abuse, residual effects, and bacterial resistance (Nielsen and Friberg, 2013; Li et al., 2017). The PK/PD model exhibits remarkable similarity to animal infection models and precisely replicates the dynamics of pharmacokinetics (Liang et al., 2013). The model can be used to eliminate the differences among animal species, revealing the real time-kill curve of the drug concentration and bacterial clearance rate (Booker et al., 2005). Undoubtedly, disparities exist between *in vitro* PK/PD models and *in vivo* animal models for mastitis infections (Meletiadis et al., 2012). The PK/PD model not only guarantees animal welfare, but also reduces experimental costs associated with mastitis in dairy cows (Yu et al., 2016). Therefore, establishing an *in vitro* dynamic model appears to be a feasible approach for evaluating the effects of CFQ against *S. uberis*.

The primary resistance mechanisms of *β*-lactam antibiotics include the β-lactam enzymes of bacteria that facilitate antibiotic inactivation; binding with β-lactam enzymes hinders the ability to access the target site (PBPs) and reduces the affinity between PBPs and antibiotics, as well as alterations in the permeability of the cell wall or outer membrane (Bryskier, 1997; Thomas et al., 2006).

The aim of this study was to establish an *in vitro* PK/PD model to simulate the pharmacokinetics of CFQ and evaluate its antibacterial activity against *S. uberis*. We compared the antimicrobial efficacy of a single high-dose regimen with that of multiple low-dose regimens and the best-fit dosage for administration. The best-fit PK/PD parameters were used to determine whether CFQ exhibited concentration-dependent or time-dependent effects against *S. uberis*. The second objective was to determine the sensitivity of bacteria to changes in their biofilm formation ability. These findings provide valuable insights for optimizing antimicrobial drug administration regimens and high-dose sustained-release preparations to reduce the action time of low drug concentrations in clinical treatment.

## 2 Materials and methods

### 2.1 Bacteria and drug

*Streptococcus uberis* S0 was isolated from clinical milk samples from cows with mastitis and stored at −20°C before use. Sulfate CFQ, obtained from the National Institutes for Food and Drug Control (Beijing, China), was also stored at −20°C prior to use. The Todd-Hewitt Broth (THB) and Todd-Hewitt Agar (THA) were purchased from Qingdao Haibo Biotechnology (Qingdao, China).

### 2.2 Inoculum preparation

*Streptococcus uberis* was resuscitated by inoculating a small aliquot of the frozen culture liquid from −20°C onto a THA plate and incubating at 37°C for 36 h. Then, 5 mL of THB medium was used to cultivate a single colony from the plate overnight until reaching the logarithmic phase. Then, 100 μL of the logarithmic growth culture was transferred to 1 mL of THB. The final concentration of the culture was approximately 10^6^ colony forming units (CFU)/mL.

### 2.3 Determination of minimal inhibitory concentration, minimal bactericidal concentration, and mutant prevention concentration

The minimal inhibitory concentration (MIC) and minimal bactericidal concentration (MBC) of CFQ against *S. uberis* were determined using the microbroth dilution method modified in accordance with CLSI. Briefly, cultures in the logarithmic growth phase were diluted with Mueller-Hinton Broth (MH) to 1 × 10^6^ CFU/mL. The bacterial suspension of 180 μL was added to the first column of the 96-well plate, and 100 μL MH broth was added to columns 2–11. CFQ (20 μL) was added to each well in the first column, and double dilution was performed. Two hundred microliters of sterilized MH broth and the bacterial suspension were added to the 12th column as negative and positive controls, respectively. The results were evaluated after incubation at 37°C for 16–20 h, and each experimental underwent replication three times. The lowest concentration of the antibiotic that inhibited visible bacterial growth was determined as the MIC. Following an overnight incubation at 37°C, a 100 μL aliquot of the bacterial suspension was carefully transferred from each well to an MH agar plate. The lowest antibiotic concentration that resulted in the absence of visible bacterial growth on the plate was defined as the MBC.

Single colonies were inoculated in 20 mL of THB overnight to reach 6–8 × 10^8^ CFU/mL and transferred to 200 mL of THB for 8 h. After centrifugation at 8000 rpm for 10 min, the bacteria were suspended in 3 mL of THB adjusted to 3.0 × 10^10^ CFU/mL. Subsequently, 100 μL of bacterial solution was plated onto THA plates containing different drug concentrations to achieve a total bacterial inoculation of 1.2 × 10^10^ CFU, and this was repeated four times. The bacterial colonies were counted at 24, 48, and 72 h until bacterial growth was observed. The mutant prevention concentration (MPC) represented the lowest drug concentration that inhibited bacterial growth after incubation for 72 h. The drug concentration was designed based on the MPC using a linear decay rate of 20%, and the anti-mutation concentration was defined as the lowest concentration without colony growth.

### 2.4 Time-kill curve studies

Eight drug concentrations were set based on the MIC within a certain range (1/2, 1, 2, 4, 8, 16, 32, and 64 × MIC). Additionally, a growth control group (without drugs) and a sterility control group (without bacteria) were included. The culture system consisted of 4 mL of sterile medium followed by the addition of 0.5 mL drug solution of 10 times the final drug concentration and 0.5 mL of bacterial suspension for a final inoculation of 10^6–7^ CFU/mL. The mixtures were cultured at 37°C for 48 h. Aliquots (100 μL) of the cultures were collected from each group at 0, 2, 4, 6, 8, 10, 12, 24, and 48 h to perform a tenfold dilution before transferring 100 μL of each sample onto agar plates. The detection limit for bacteria was set at 10 CFU/mL, and the experiments were conducted in triplicate. The logarithmic value of the counting (log_10_ CFU/mL) was taken as the ordinate, and time was taken as the horizontal coordinate to draw the sterilization curve.

### 2.5 Description of the model

**Figure 1:**
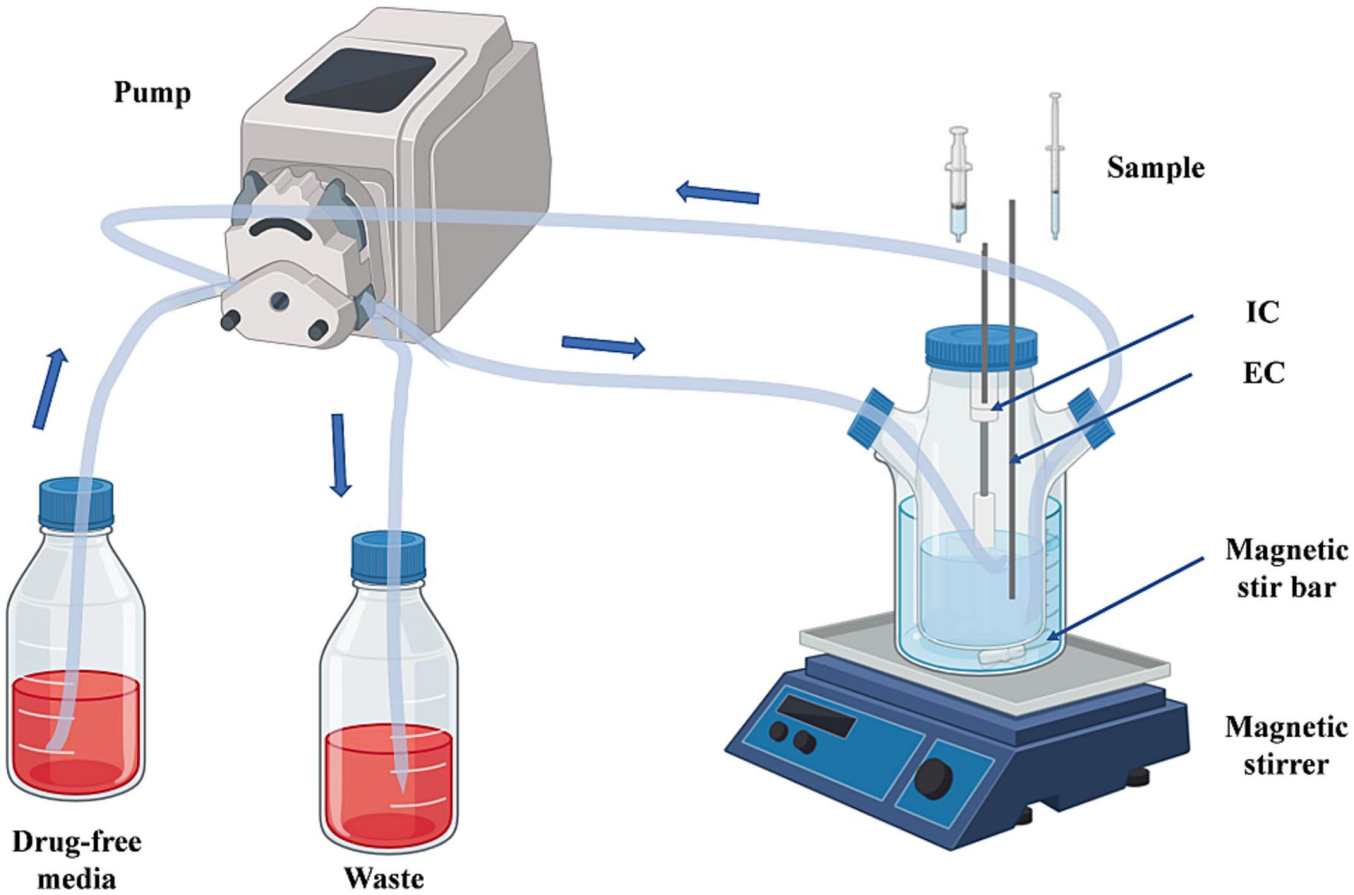
The *in vitro* dynamic model simulates the pharmacokinetics of cefquinome and the bactericidal effect and susceptibility of *S. uberis*. EC, external compartment; IC, internal compartment.

In this study, we modified the *in vitro* PD model described by Huang et al. (2019) to a one-compartment open model of CFQ (Huang et al., 2019). The model was applied in accordance with the flowchart shown in Figure 1. In brief, the model system encompasses a multichannel peristaltic pump to provide power and a thermostatic water bath with magnetic stirrers to maintain the temperature of bacterial growth. The model consisted of three compartments, one of which was a storage chamber containing 2 L of sterile medium. The second compartment was the central chamber, and the external compartment (EC) consisted of 800 mL of sterile medium and 20 mL of a semipermeable cellulose membrane (internal compartment, IC). The two chambers were diluted continuously using a peristaltic pump. The third chamber was an empty bottle used to collect the waste. The thermostatic water bath maintained the temperature of *S. uberis* (37°C) and was used to mix the drug and medium. The operation of the peristaltic pump provided a constant rate of change in the drug concentration over time. The experiment was based on a precise simulation of drug concentrations and the parameters determined in our laboratory (unpublished). The flow rate of the peristaltic pump was set at 0.7 mL per minute.

### 2.6 *In vitro* model and dosing regimens

In this study, we aimed to validate the therapeutic efficacy of single high-dose and multiple low-dose administrations based on previous pharmacokinetic *C*_max_ data. To achieve this, we established five groups for single-dose regimens (0.28, 0.5, 1, 1.13, and 1.5 mg/L) and three groups for multiple doses (0.09 mg/L, 3; 0.5 mg/L, 2; 0.25 mg/L, 4 and all at intervals of 24 h). At time zero, CFQ was injected into the central chamber and membrane with 15 mL *S. uberis* at 10^6^–10^7^ CFU/mL. The magnetic stirrer accelerated the speed to achieve a balance between the intramembrane and extramembrane drug concentrations.

Samples (0.5 mL) were collected from the EC at 0.15, 2, 4, 6, 8, 10, 12, 24, 36, 48, 72, 96, and 120 h after administration and then stored at −20°C until analysis. Samples (100 μL) were taken from the IC 0, 2, 4, 6, 8, 10, 12, 24, 36, 48, 72, 96, 120, and 144 h after administration. The number of *S. uberis* and its susceptibility were determined using the collected samples.

### 2.7 Detection of CFQ

The concentration of CFQ in the medium was analyzed using high-performance liquid chromatography (HPLC). Because of the relatively low protein binding rate of CFQ, acetonitrile was chosen as the extractant. The sample (20 μL) was injected after filtration through a 0.22 μm filter. The composition of the mobile phase was as follows: Solution A (0.025 mol/L sodium perchlorate solution): solution B (acetonitrile): phosphoric acid = 1,000:105:12, at a flow rate of 1 mL/min. The detection wavelength was 270 nm, and the column temperature was 30°C. The PK parameters were calculated using DAS 2.1 (Drug and Statistics) software.

### 2.8 Time-killing curve fitting and analysis

The antibacterial efficacy of drugs can be evaluated based on their kill rate. The greater the kill rate, the stronger the bactericidal effect of the drug on bacteria (Nielsen et al., 2007; Huang et al., 2019). Linear regression was used to determine the slopes at different time intervals (0–24, 0–48, 4–24, 4–48, and 8–48 h) as the bactericidal rates. Linear regression analysis was performed considering the overall situation at different points throughout the time interval. The relationship between the average kill rates at five times intervals and each concentration was analyzed using the *E_max_* model in DAS 2.1. The *E_max_* model can be described using the following formula:


$$E=\frac{E_{\max}\times C_{e}^{N}}{EC_{50}^{N}+C_{e}^{N}}$$

Where *E* is the kill rate, *E_max_* is the maximum kill rate, C_e_ is the CFQ concentration, *EC_50_* is the CFQ concentration when the kill rate is half the maximum value, and N is the Hill coefficient, which is used to reflect the slope of the curve. *R*^2^ reflects the degree of concordance between the experimental data and the predictions made by the *E_max_* model, where a higher R^2^ value indicates a superior fit for these data, suggesting a strong correlation between the kill rate and drug concentration.

### 2.9 Susceptibility testing of *S. uberis*

The sample was collected at the last point and inoculated into fresh culture medium overnight until it reached the logarithmic phase. The MIC was determined using the microbroth dilution method, as described in the Determination of the Minimum Inhibitory Concentration section.

### 2.10 Pharmacokinetic-pharmacodynamic integration

By integrating the PK parameters and the MBC value *in vitro*, two important PK/PD indices, %T > MBC and AUC_72h_/MBC, were calculated. The inhibitory *E_max_* model was utilized to fit the correlation between the PK/PD parameters and antibacterial effects. The R^2^ value was employed as a measure of the correlation between the PK/PD parameters and antibacterial effect, where a higher value indicates a stronger correlation. The formula is as follows:


$$E=E_{\max}-\frac{(E_{\max}-E_{0})\times C_{e}^{N}}{EC_{50}^{N}+C_{e}^{N}}$$

where *E* is the antibacterial effect, *E_max_* is the change in the population of bacteria in the control group with no treatment, *E_0_* is the largest bacterial decrease in the treatment group, *C_e_* represents the PK/PD indices (%T > MBC, AUC_0-72h_/MBC), *EC_50_* is the corresponding PK/PD index value when the antibacterial effect reaches 50% of the maximum antibacterial effect, N is the Hill coefficient that describes the slopes of the PK/PD index-effect curve, and *R*^2^ was calculated for each assay.

### 2.11 Detection of biofilm formation ability

Samples were collected at the last time point and inoculated overnight with THA. Individual colonies were picked, cultured to the logarithmic phase, and inoculated into THB containing 1% glucose at a concentration of 1 × 10^7^ CFU/mL. Then, 200 μL of the inoculum was transferred to 96-well polystyrene plates, with each strain replicated in three wells. The medium served as a blank control, and the plates were incubated at 37°C for 48 h. The medium was discarded, and the plates were washed three times with PBS to remove planktonic bacteria. Subsequently, 200 μL of methanol was added to fix the biofilm for 15 min. After discarding the methanol and allowing the plates to dry, 200 μL of 0.1% crystal violet was added for staining for 5 min, and the plates were dried again. Then, 200 μL of absolute ethanol was added to determine at ODC_595_ after high-speed oscillation for 20 min. Blank wells without inoculated strains served as blank controls. The experiment was repeated three times. The statistical analysis method used was one-way ANOVA.

## 3 Results

### 3.1 MIC, MBC, and MPC

The MIC value of CFQ against *S. uberis* S0 was 0.0156 μg/mL, using the microdilution method. The MBC and MPC values were 0.125 and 0.02 μg/mL, respectively.

### 3.2 Time-killing curve fitting and analysis

**Figure 2:**
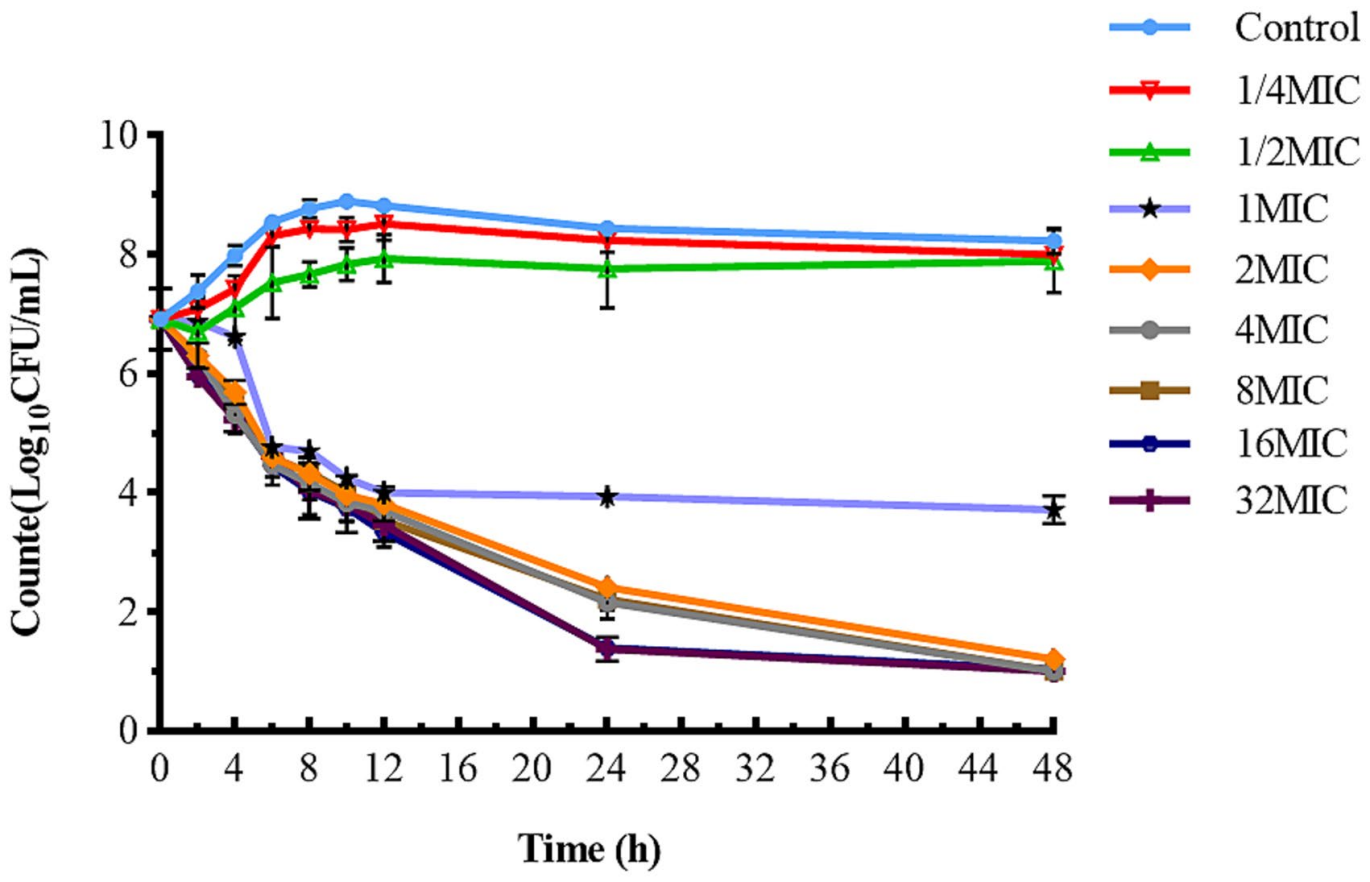
Time-killing studies of cefquinome against *S. uberis* at different concentrations. Data are presented as geometric means of three experiments. MIC, minimum inhibitory concentration; CFU, colony forming units.

The static time-kill curve is illustrated in the scatterplot shown in Figure 2. CFQ demonstrated significant antibacterial activity against *S. uberis* when its concentration exceeded 1 × MIC, whereas it exhibited poor antibacterial activity when the concentration was below 1 × MIC. At CFQ concentrations of 1/4 × MIC and 1/2 × MIC, there was only a marginal decrease by 0.31 and 0.52 log_10_ (CFU/mL), respectively, compared to the control at 48 h. When CFQ was exposed to 1–32 × MIC, a substantial reduction was observed at 48 h, ranging from 3.31 to 6.13 log_10_ (CFU/mL), and antibacterial effects were achieved. The antibacterial effect against *S. uberis* did not intensify with increasing drug concentrations, which was consistent with its time-dependent characteristics. Undoubtedly, the number of colonies decreased to the lowest detection limit (10 CFU/mL) at concentrations ranging from 2 to 32 × MIC at 48 h.

**Figure 3:**
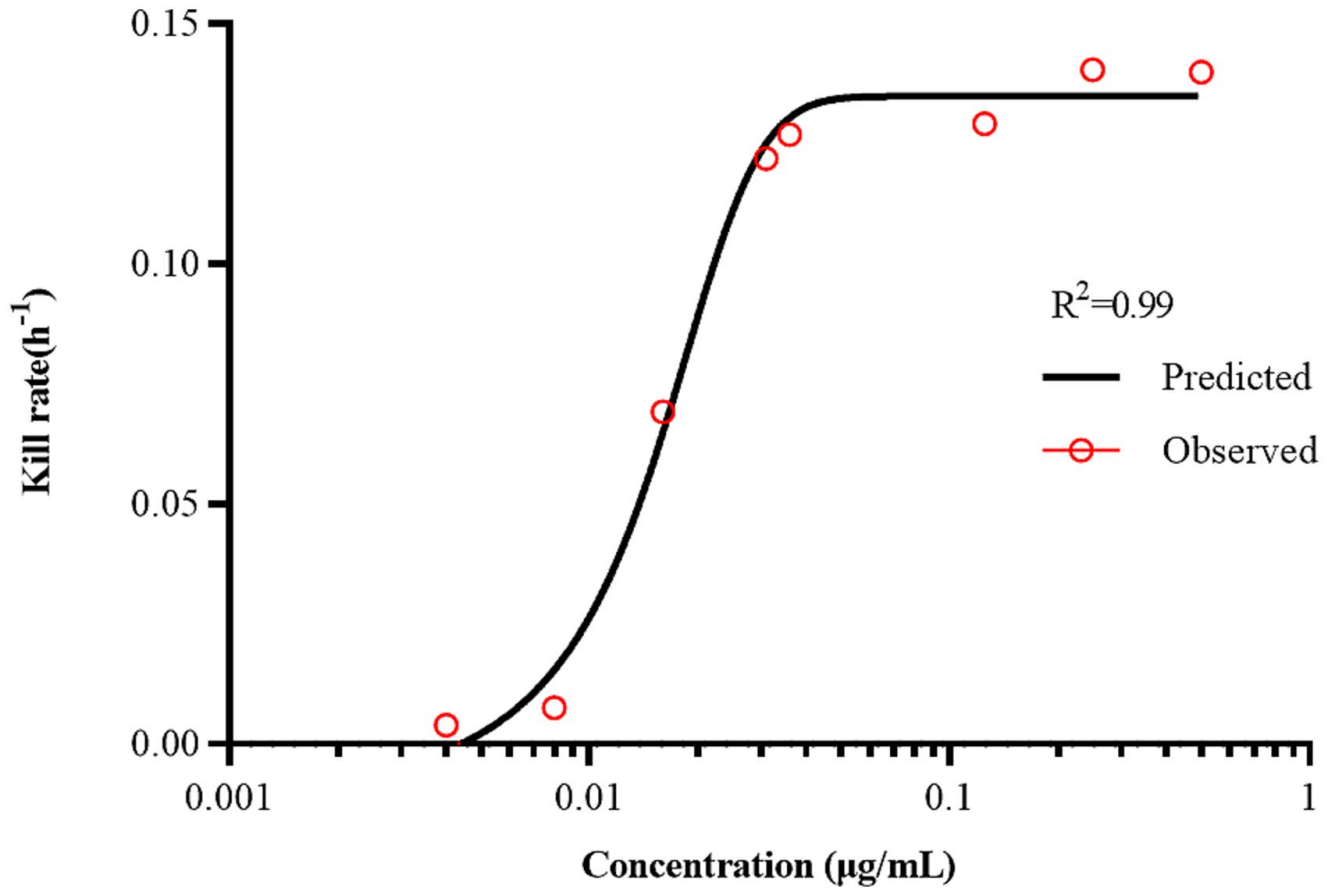
The best-fit curve obtained from the *E_max_* model of static time-killing assay data. *R*^2^ is the correlation coefficient.

The antibacterial effect of a drug can be evaluated based on its kill rate. Therefore, we fitted the data with the *E_max_* model based on the kill rate and concentration. The fitting curve between the drug concentration and kill rate is shown in Figure 3 (the time interval was set at 0–48 h). To reduce errors, the kill rate at five times intervals was selected to represent the slope of the time-kill curve (Regoes et al., 2004; Ferro et al., 2015). The kill rate increased with increasing drug concentration and became increasingly constant as the concentration increased. The kill rate in the first 24 h was relatively rapid, the maximum kill rate was 0.22 h^−1^, and the correlation between the kill rate and concentration was the highest (*R*^2^ = 0.99). The parameters fitted using the *E_max_* model are listed in Table 1.

**Table 1:** The kill rate parameters derived from the *E_max_* model of static time-killing assay data.

*E_max_* model parameter	Value
*R* ^2^	0.99
*EC_50_*	0.02
*E_max_*	0.14
Hill’s slope	3.45

### 3.3 Bactericidal effects in the *in vitro* dynamic model

**Figure 4:**
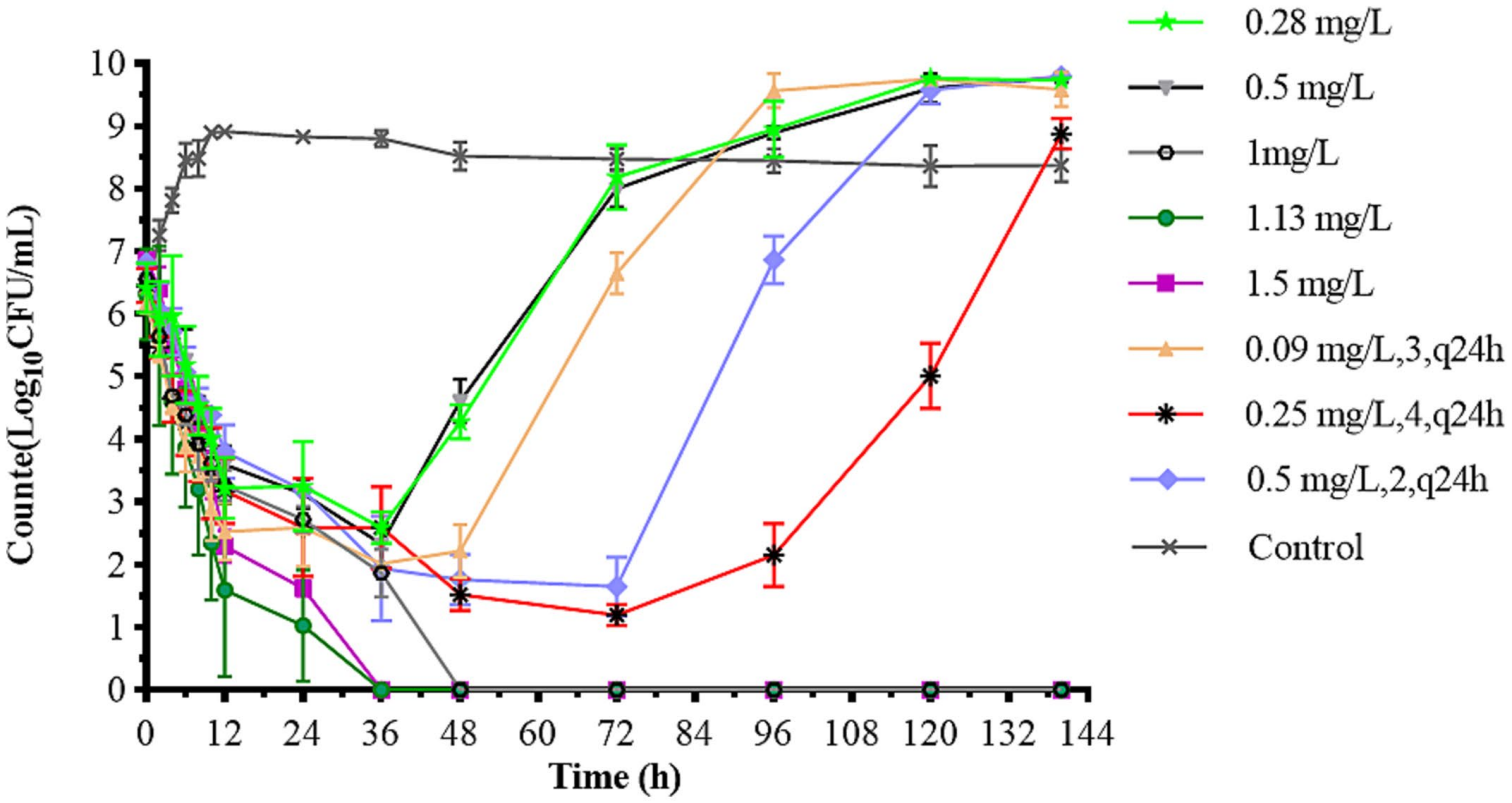
Dynamic time-killing curves depicted at two dosage regimens. Data points represent geometric means of three experiments.

Figure 4 illustrates the antibacterial effects of varying doses of CFQ against *S. uberis in vitro* using a dynamic model. Bactericidal activity increased as the drug concentration decreased below 1 mg/L in single-dose administration. After 144 h of treatment, single-dose regimens at 1–1.5 mg/L eliminated bacteria at 24–36 h, and the bacteria no longer regrew when were transferred to fresh medium for 48 h or longer. In the single-dose groups of 0.28 mg/L and 0.5 mg/L, significant bacterial regrowth to 9 log_10_ (CFU/mL) was observed after a reduction by 3.82–4.23 log_10_ (CFU/mL) within 36 h. All multiple dosage regimens also resulted in notable bacterial regrowth after a reduction. The multiple-dose groups showed a decrease to below 2 log_10_ (CFU/mL) at 48 h, even below the detection limit, but also exhibited an increase to 9 log_10_ (CFU/mL) at 120 h. But the group of 0.25 mg/L (4, q24) increased at 144 h. The maximum bacterial count of *S. uberis* decreased by 3.82 log_10_ CFU/mL to all eliminated in all groups, achieving bactericidal activity against *S. uberis*. The bactericidal effect of a single high dose was better than that of multiple low doses. When the bacterial populations were reduced to a certain number, there was a lag time at which the population did not change significantly within the multi-low-dose treatments, with a lag time of 36 h for 0.09 mg/L, 3 and 0.5 mg/L, 2; 72 h for 0.25 mg/L, 4 group, respectively.

### 3.4 *In vitro* simulated pharmacokinetics

**Figure 5:**
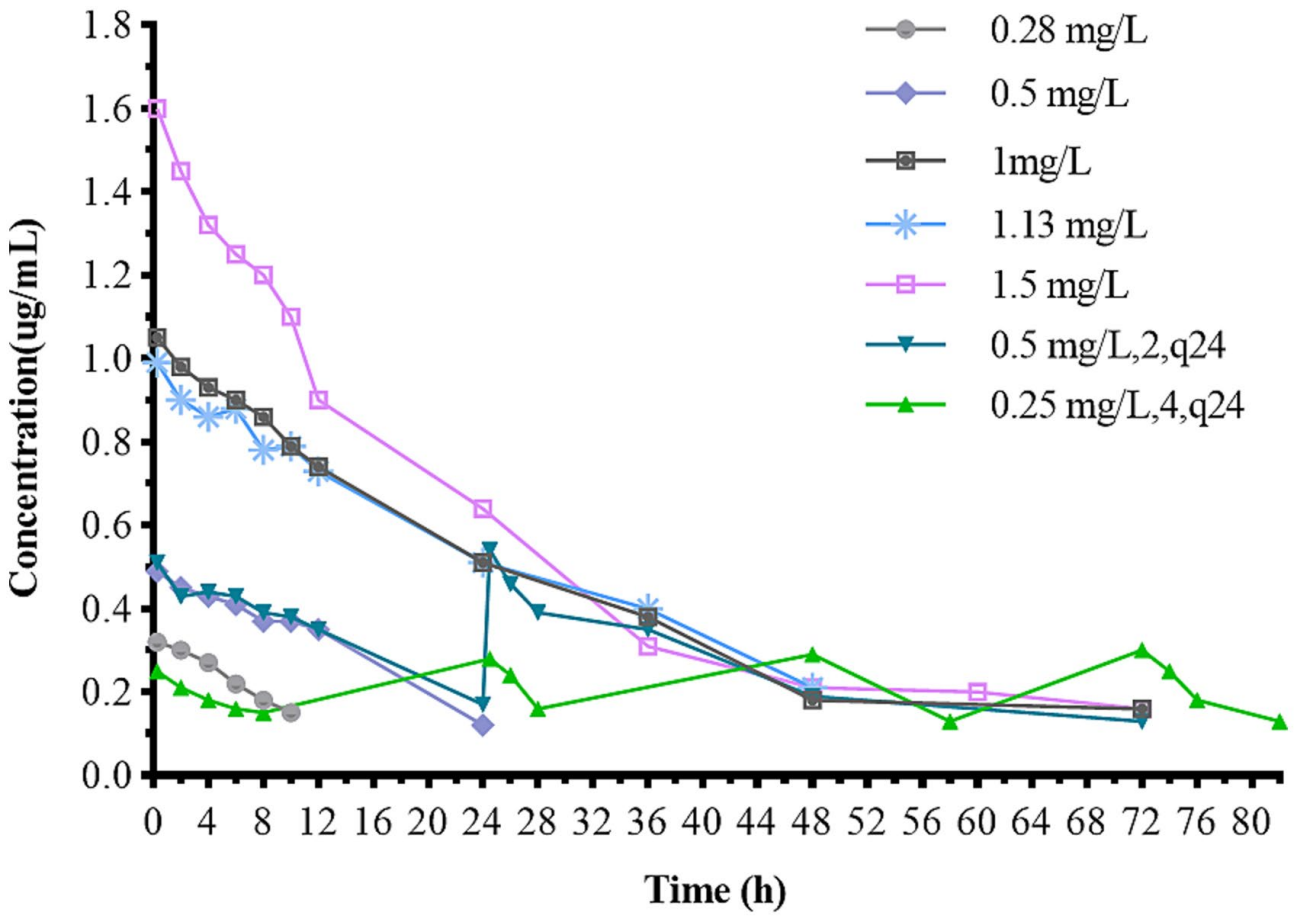
Concentration-time curves of cefquinome in the *in vitro* dynamic model. The dosage regimen of 0.09 mg/L (3, q24) is not presented because the concentration is below the limit of quantification.

The pharmacokinetic time-concentration curves for different doses are shown in Figure 5. Table 2 summarizes the PK parameters. The one-compartment open model highly correlated with the data from each dose group (*R*^2^ > 0.99).

**Table 2:** Main pharmacokinetic parameters of different dosages in the *in vitro* PK/PD model.

Pharmacokinetic parameters	Dose group (mg/L)							$\bar{X}$
	0.28	0.5	1	1.13	1.5	0.25,4, q24h	0.5,2, q24h	
Total dose (mg)	4.2	7.5	15	16.9	22.5	15	15	
*t*_1/2β_ (h)	14.63	12.18	18.05	16.22	15.84	15.15	17.28	15.62
*V*_(central)_ (mL)	810	810	820	800	850	810	820	817.14

The pharmacokinetic parameters of the dosage regimen of 0.09 mg/L (3, q24) are not presented because the concentration was below the limit of quantification.

### 3.5 Susceptibility testing of *S. uberis*

Five different strains were isolated from various dose groups after 144 h, except for the 1, 1.13, and 1.5 mg/L groups. We renamed the surviving bacteria from different dosage groups in the PK/PD model as S1–S5 to facilitate subsequent experiments. S1 (0.28 mg/L), S2 (0.5 mg/L), S3 (0.09 mg/L, 3), S4 (0.5 mg/L, 2), and S5 (0.25 mg/L, 4) strains were selected, respectively. The susceptibility to CFQ was assessed, and the alteration of the MIC of S1–S5 is presented in Table 3. The MIC of S1–S5 for CFQ ranged from 0.0156 to 0.0625 μg/mL. Compared to the original MIC values, S1–S4 exhibited no changes and did not demonstrate any development of resistance. However, a slight increase in MIC to 0.0625 μg/mL was observed for S5, which represented a four-fold increase from the original value but remained below the resistance break point far away.

**Table 3:** The MIC of cefquinome against *S. uberis* S1–S5 strains.

Strain	S1	S2	S3	S4	S5
MIC (μg/mL)	0.015625	0.015625	0.015625	0.015625	0.0625

S1 (0.28 mg/L), S2 (0.5 mg/L), S3 (0.09 mg/L, 3), S4 (0.5 mg/L, 2), S5 (0.25 mg/L, 4) strains were selected from different dosages, respectively.

### 3.6 Pharmacokinetic/pharmacodynamic modeling and analysis

**Figure 6:**
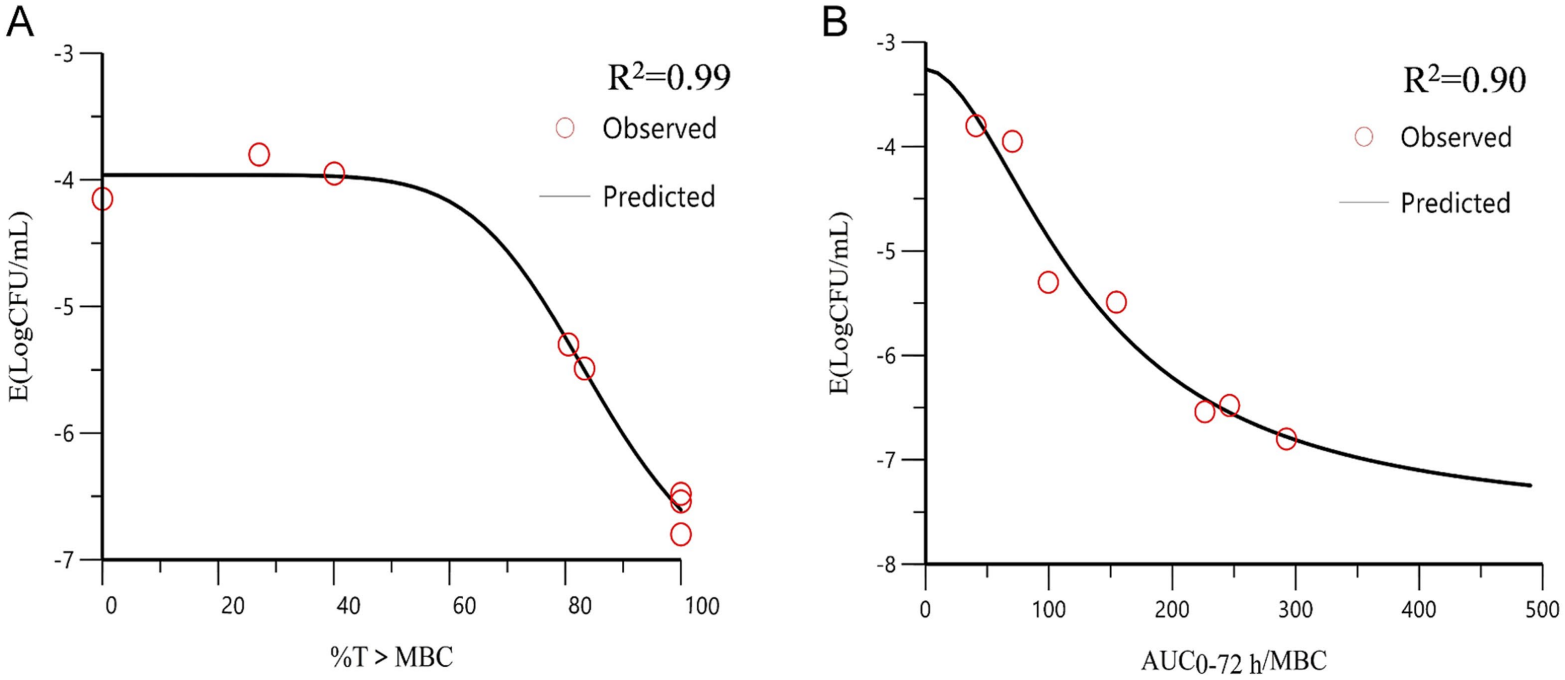
The curve of the PK/PD parameters versus the antibacterial effect. **(A)** %T > MBC-antibacterial effect curve; **(B)** AUC_0-72h_/MBC-antibacterial effect curve. *R*^2^ is the correlation coefficient. AUC, area under the concentration-time curve; %T > MBC is the time percentage that the concentration exceeds the MBC.

We quantified antibacterial efficacy using the logarithmic reduction in bacterial counts (log_10_ CFU/mL). The correlation between the PK/PD parameters and antibacterial efficacy is shown in Figure 6. The correlation coefficients for AUC_0-72h_/MBC, %T > MBC, and antibacterial effects showed good linear relationships, which were determined to be 0.90 and 0.99%, respectively. The distribution of %T > MBC was wide and covered the entire percentage range. These findings suggest that %T > MBC serves as the best-fit PK/PD parameter for predicting the antibacterial activity of CFQ against *S. uberis*, which displayed a time-dependent nature. Table 4 lists the key parameters, including *E*_0_, *E*_max_, *EC*_50_, and the Hill coefficient.

**Table 4:** Estimation of PK/PD parameters from the *E_max_* model.

PK-PD parameter	*E_max_* (log_10_CFU/mL)	*EC_50_*	*E_0_* (log_10_CFU/mL)	Hill’s slope	*R* ^2^
AUC_0-72h_/MBC (h)	−5.72	147.08	−2.78	1.26	0.90
T > MBC (%)	−3.44	85.62	−3.96	7.73	0.99

### 3.7 Detection of biofilm formation ability

**Figure 7:**
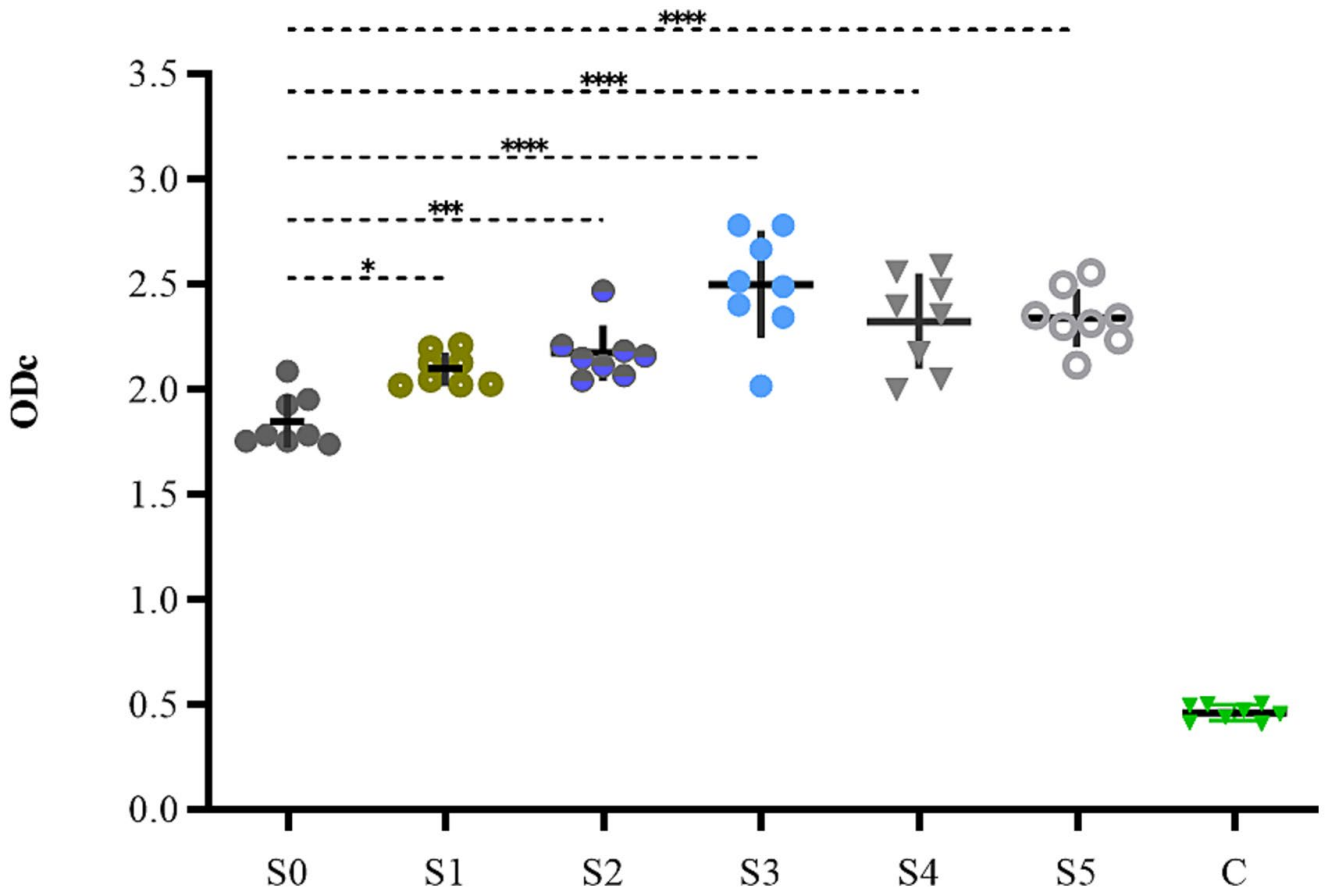
The biofilm formation ability of S1–S5.

The biofilm-forming abilities of the obtained strains were examined, and the results are shown in Figure 7. The biofilm-forming abilities of S3, S4, and S5 increased from medium to strong, showing extremely high significance (*p* < 0.0001). The biofilm formation ability of S1 and S2 showed slight enhancement with significant difference, having *p* values of <0.05 and <0.001, respectively. However, the actual magnitude of variation in their biofilm formation was relatively small, and both strains maintained medium forming ability.

## 4 Discussion

*Streptococcus uberis* can persist in the mammary gland, causing infections in various bovines and is the most common and difficult environmental pathogen to eradicate (Sharun et al., 2021). β-lactam antibiotics are commonly used clinically to treat streptococcal mastitis. Antimicrobial activity is typically assessed on the basis of a series of clinical symptoms or pathological changes (Thomas et al., 2006; Gloede et al., 2010). However, no information is currently available on the PK/PD interactions of CFQ against *S. uberis*.

**Figure 8:**
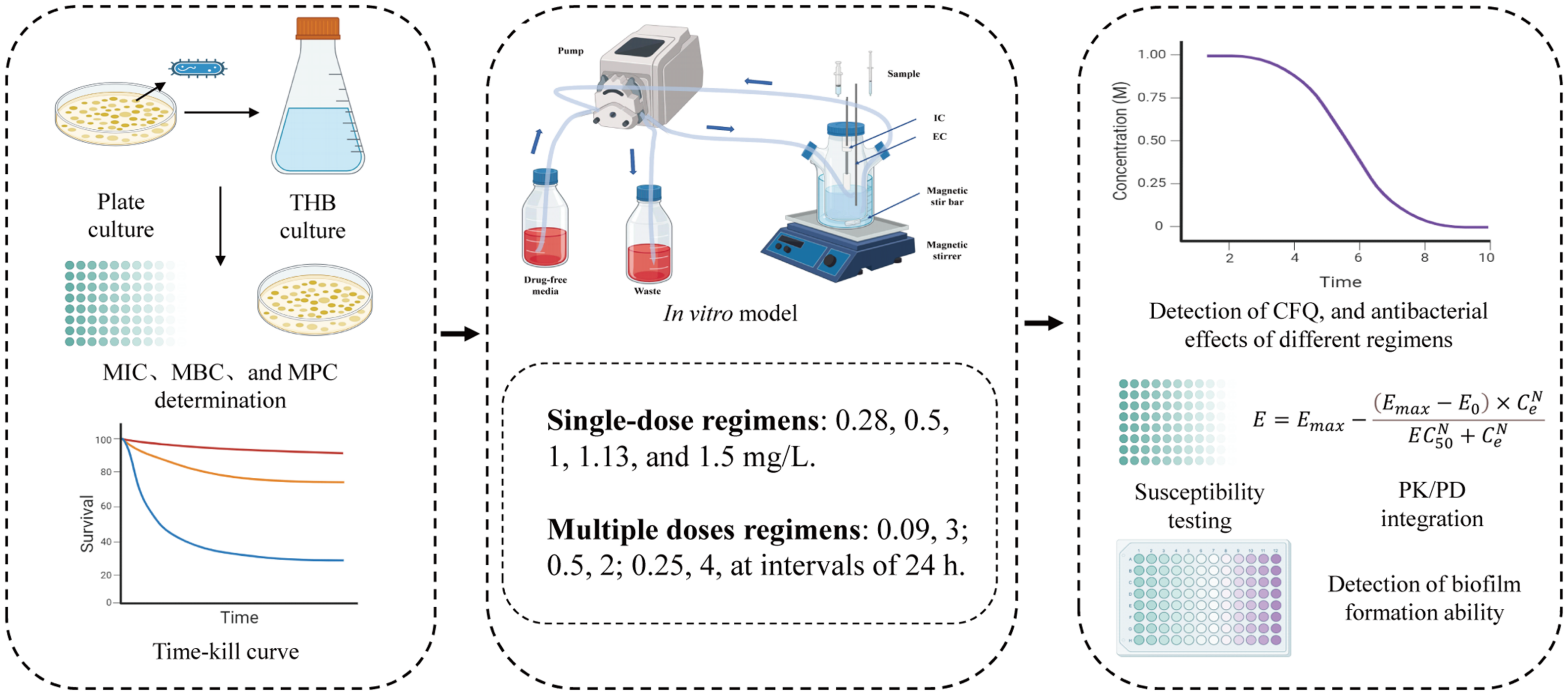
The flowchart of the main procedures in the study.

The PK/PD models, including AUC/MIC and % T > MIC, have been studied for antibiotics for many years (Toutain and Lees, 2004). Presently, the central compartment mainly consists of semipermeable cellulose membranes and hollow fiber models, which maintain a constant volume and drug can freely permeate and prevent bacteria from pumping out of the central compartment (Gumbo et al., 2007; Meletiadis et al., 2012). Huang et al. established a first-order absorption one-compartment open model to reveal the PK/PD relationship of tilmicosin and *Mycoplasma gallisepticum in vivo* for studying bactericidal effects and resistance of different dosage regimens (Huang et al., 2019). An intravenous one-compartment model combined with PK and a mutation selection window (MSW) was employed to study the effects of different drug doses on bacterial killing rates and resistance (Firsov et al., 2003). Previously, PK/PD modeling of CFQ against mastitis was investigated in a mouse mastitis model (Krone et al., 2007). In this study, we adopted a semipermeable cellulose membrane considering the sterilization of the device, simplicity, and repeatability of the operation. The present study is the first to use an *in vitro* peristaltic pump model to evaluate the PK/PD relationship of CFQ against *S. uberis*, which can monitor drug concentration over time, antibacterial effects, and sensitivity. The advantage of the model is that when it is difficult to establish a dairy mastitis model, the therapeutic effects of CFQ against *S. uberis* can be compared using two different dosing regimens to determine the best-fit PK/PD parameters. Changes in the biofilm formation ability were determined. Figure 8 illustrates the main procedures of the present study.

**Figure 9:**
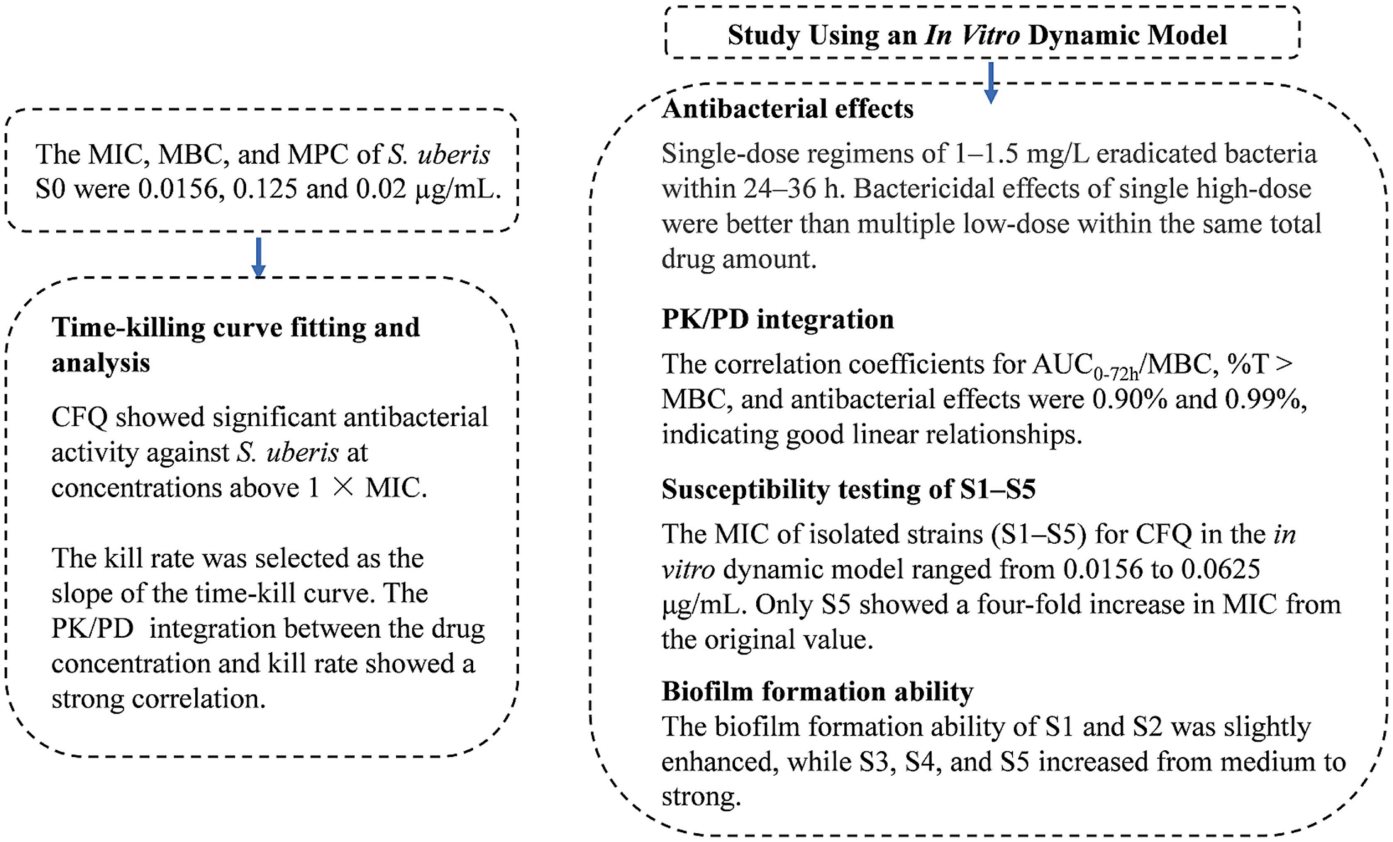
The main results of the study.

The main results of the present study are presented in Figure 9. In the study, the MIC of strain S0 that was sensitivity to CFQ was 0.0156 μg/mL. The survey of the susceptibility of bacteria causing mastitis in Europe and North America from 1990 to 2002 showed that the MIC of CFQ for *S. uberis* was 0.03–0.25 μg/mL, with MICs < 0.03–0.13 μg/mL for *S. agalactiae* and <0.008–0.25 μg/mL for *S. dysgalactiae*. All of these MICs were lower than that of *S. aureus* (0.5–1 μg/mL) (Ehinger et al., 2006). The MICs of strain S0 were similar to those reported by Anno de Jong, where the MICs of *S. uberis* for CFQ were 0.008–0.5 μg/mL. The sensitivity of *S. uberis* for CFQ was lower than that for ceftiofur (0.12–2 μg/mL); both drugs are commonly used for treating clinical mastitis (de Jong et al., 2018). The MIC of *S. uberis* for ceftiofur was ≤0.06–8 μg/mL (Sweeney et al., 2024). The MPC of strain S0 was 0.02 μg/mL, and the SI (selection index, MPC/MIC) being 1.28 indicates that the MSW range was narrow; drug concentrations exceeding the MPC can prevent the enrichment of resistant bacteria. In Zhang’s study, the MIC and MPC of CFQ against *Escherichia coli* were 0.064 and 0.16 μg/mL, respectively, with an SI of 2.5, which is similar to the results of our study (Zhang B. et al., 2014).

The MBC of S0 was 0.125 μg/mL, which is higher than the MIC and MPC, and different from the results obtained from other studies. The MIC of *P. multocida* and *E. coli* were 0.02 μg/mL and 0.05 μg/mL, respectively, while the MBC was 0.05 μg/mL and 0.06 μg/mL, with MIC close to MBC (Dinakaran et al., 2013). Similar results were shown in Shan’s study (Shan et al., 2014). The MIC, MBC, and MPC of CFQ against *S. aureus* were 0.25, 0.5, and 4 μg/mL, respectively, with MPC higher than MBC and SI of 16, which was significantly different from that of strain S0 (Ahmad et al., 2015). However, there has been relatively limited research on the effects of CFQ on *S. uberis* MBC and MPC.

%T > MIC and AUC_0-24h_/MIC were the main PK/PD parameters of β-lactam antibiotics (Craig, 1998a; Andes and Craig, 2002; Zhang B. X. et al., 2014). Some studies have reported the antibacterial activity of CFQ as %T > MIC (Shan et al., 2014; Zhao et al., 2017). Antibacterial effects can be achieved in severe cases and in immunodeficient animals when the %T > MIC is above 80% of the dosing interval (Craig, 1998b; Toutain et al., 2002). Corum et al. (2019a) demonstrated similar results for sensitive bacteria. In the present study, we chosen MBC as the PD parameter for evaluating the bactericidal effect of CFQ. The reasons were as follows: the MBC of strain S0 was measured at 8 × MIC and 6.25 × MPC in the study. Time-dependent sterilization depends on the length of exposure, where maintaining %T > MIC at 100% requires 4–5 times the MIC (Corum et al., 2019b). Maintaining drug concentrations outside the mutant selection window (MSW) or above the MPC can effectively suppress the emergence of resistant mutants while enhancing bactericidal efficacy (Zhao and Drlica, 2001). Integrating MBC with PK parameters to establish a PK/PD model enables prediction of the optimal dosing regimen required to achieve bactericidal effects, maximize therapeutic efficacy, and minimize resistance development. The results of the present study showed that AUC_0-72h_/MBC and %T > MBC had the highest correlation with antibacterial effects, reaching 0.90 and 0.99%, respectively. The %T > MBC was the best-fit PK/PD parameter, suggesting that CFQ had time-dependent activity. The AUC_0-24h_/MBC and *C*_max_/MBC parameters were used to evaluate the antibacterial effects of CFQ against *S. aureus* (Piscitelli et al., 1992; Ahmad et al., 2015). Huang et al. (2019) established an *in vitro* PK/PD model to evaluate the bactericidal effect of tilmicosin on *M. gallisepticum*; the correlation coefficients of AUC_0-24h_/MIC and %T > MIC were 0.87 and 0.49%, respectively. When the concentration of the drug is high enough to show bactericidal activity, it may result in the high correlation between AUC_0-72h_/MBC and the bactericidal effect of CFQ against *S. uberis*. In our previous study, we administered a sustained-release injection of CFQ to cows via intramuscular injection, which was developed in the laboratory. The PK results showed that CFQ was eliminated slowly and led to a drug concentration above the MBC for 30 h to maintain the effective antibacterial concentration. Therefore, antibacterial activity is determined by the characteristics of the drug and the bacteria (Piscitelli et al., 1992).

All the dose groups in this study exceeded the MPC, which may have prevented the selection of resistant strains. The sensitivity of strains S1–S5 remained relatively stable, except for strain S5 (0.0625 μg/mL). A possible reason for this might be that the MBC is higher than the MPC, resulting in a drug concentration higher than that of the MPC during the dosing interval. Thus, high-dose administration in clinical treatment can enhance therapeutic efficacy and prevent resistance to mutations.

The static bactericidal curve indicated that there was no bactericidal effect at 1/4× and 1/2× the MIC. The bacteria decreased by 3.31 log_10_ (CFU/mL) at 1× the MIC, whereas a decrease of 5.98 log_10_ (CFU/mL) was observed after 48 h when the concentrations exceeded 2 × MIC. Moreover, the bactericidal effect did not increase with increasing concentration. Figure 4 showed that the dosage groups of 1, 1.13, and 1.5 mg/L eliminate bacteria in the dynamic model. To confirm whether *S. uberis* was completely eradicated, the medium within the cellulose membrane was transferred to a fresh medium for further culture, and no regrowth was observed. However, re-growth was observed within 96 h in the single-and multiple-dose groups. Notably, the groups of 0.28 mg/L and 0.5 mg/L demonstrated a reduction of 3.82 and 4.83 log10 (CFU/mL) at 36 h, respectively, followed by a sharp increase to 9 log_10_ (CFU/mL) at 120 h. The multiple-dose groups decreased to below 2 log_10_ (CFU/mL) at 48 h, but also increased to above 9 log_10_ (CFU/mL) at 120 h, but the group of 0.25 mg/L (4, q24) increased at 144 h. Interestingly, as the frequency of administration increased, the lag time after the population decrease increased, followed by exponential growth. The main reasons for this are as follows: first, the drug was gradually diluted to approximately its MIC. Second, the persistence and tolerance of *S. uberis* occurred under the continuous action of CFQ. Tolerance and persistence represent analogous phenomena in the presence of antibiotics without a corresponding increase in the MIC. Persistence refers to the capacity of a subset of a population to survive exposure to bactericidal drug concentrations, whereas tolerance is the general ability of a population to survive longer treatments (Balaban et al., 2019). However, the specific mechanisms underlying the phenomenon of lag time were not investigated in this study. In contrast to Huang’s study, which reported the emergence of resistant mutant strains, no resistant strains were observed in this study (Huang et al., 2019). Interestingly, the biofilm-forming capacity of S1–S5 was enhanced, with S1 and S2 exhibiting slight increases, whereas S3–S5 displayed a more pronounced transformation from moderate to strong membrane formation. This phenomenon may be related to the expression of related genes, and resistance could be influenced by the complex environment and actual conditions of the host animal.

Previous studies primarily utilized mouse mastitis models to study the PK/PD of CFQ (Jiang et al., 2022). We first reported the PK/PD of CFQ against *S. uberis* using an *in vitro* dynamic model. CFQ is characterized by low protein binding and rapid absorption kinetics, with a plasma protein-binding rate of approximately 11% in calves, thereby reducing its influence in an *in vitro* dynamic model (Ahmad et al., 2015). The maximum total dose administered to the multi-dose groups was 1 mg/L, and comparisons were made between a single dose of 1 mg/L. The bactericidal efficacy of the single high-dose group surpassed that of multiple doses. Thus, there was no design for higher concentrations in the multi-dose groups. This study was conducted under optimal *in vitro* conditions and did not consider the potential impact of animal immune responses. Although there are inherent differences between *in vitro* PK/PD models and mastitis models in target animals, this can ensure animal welfare and reduce research costs (Gloede et al., 2010).

In conclusion, the present study demonstrated that a single administration of CFQ at 1 mg/L yields the maximum antibacterial effect against *S. uberis*, resulting in bacterial eradication without an increase in efficacy with increasing concentrations. The groups receiving two doses of 0.5 mg/L (2, q24) and four doses of 0.25 mg/L (4, q24) have equivalent total drug dosages to the 1 mg/L group, but the bactericidal effects differ markedly. The enhanced biofilm formation of S1 – S5 observed in the dynamic model suggests that prolonged exposure to drug concentrations below the MIC can lead to alterations in bacterial populations. Consequently, in clinical treatment, it is advisable to minimize the contact time between low-concentration antibiotics to mitigate the risk of resistance mutations, as well as the lag time was declined to avoid bacterial recurrence. The analysis of killing rates provides precise quantification of CFQ against *S. uberis* and confirms its time-dependence compared with using the MIC alone. Furthermore, our study revealed that the best-fit PK/PD parameter was %T > MBC (*R*^2^ = 0.99). The effective bacteria clearance could be achieved within 2 days when using the simulated dose groups of ≥1 mg/L.

The present study is the first to utilize an *in vitro* peristaltic pump model for evaluating the PK/PD relationship of CFQ against *S. uberis*. Our study showed that a single high-dose regimen demonstrated superior bactericidal effects to multiple low-dose regimens at equivalent total dosages. It effectively prevents both the extension of the lag time and the enhancement of biofilm formation capability. In PK/PD analysis, with %T > MBC being the best-fitting parameter of antimicrobial efficacy. The results demonstrated that single high-dose therapy effectively prevents relapse and reduces the development of resistant bacteria. These data provide strong support for *in vivo* studies and may facilitate the development of rational treatment strategies for *S. uberis* infections and novel sustained-release preparations. But there are inherent differences between *in vitro* PK/PD models and mastitis models in target animals. The maximum total dose administered in the multiple-dose groups was limited to 1 mg/L, and the antibacterial effects of multiple-dose regimens with higher total doses were not investigated. Additionally, the mechanisms behind lag time extension and enhanced biofilm formation remain unclear and require further study. In contrast to other studies, no resistant strains emerged in our experiments.

## Data Availability

The original contributions presented in the study are included in the article/supplementary material, further inquiries can be directed to the corresponding author.
